# A defect in cell wall recycling confers antibiotic resistance and sensitivity in *Staphylococcus aureus*

**DOI:** 10.1016/j.jbc.2022.102473

**Published:** 2022-09-09

**Authors:** Stephanie Tan, Kelvin Cho, Justin R. Nodwell

**Affiliations:** Department of Biochemistry, University of Toronto, Toronto, Ontario, Canada

**Keywords:** antibiotic resistance, peptidoglycan, cell wall, peptidoglycan recycling, walKR, MRSA, *Staphylococcus aureus*, AM, amidase domain, ddH_2_O, double-distilled water, GM, glucosaminidase domain, MIC, minimal inhibitory concentration, MRSA, methicillin-resistant *Staphylococcus aureus*, PBP, penicillin-binding protein, PP, pentapeptide, TSB, tryptic soy broth, VISA, vancomycin-intermediate *Staphylococcus aureus*

## Abstract

WalKR is a two-component system that is essential for viability in Gram-positive bacteria that regulates the all-important autolysins in cell wall homeostasis. Further investigation of this essential system is important for identifying ways to address antibiotic resistance. Here, we show that a T101M mutation in *walR* confers a defect in autolysis, a thickened cell wall, and decreased susceptibility to antibiotics that target lipid II cycle, a phenotype that is reminiscent of the clinical resistance form known as vancomycin intermediate-resistant *Staphylococcus aureus*. Importantly, this is accompanied by dramatic sensitization to tunicamycin. We demonstrate that this phenotype is due to partial collapse of a pathway consisting of autolysins, AtlA and Sle1, a transmembrane sugar permease, MurP, and GlcNAc recycling enzymes, MupG and MurQ. We suggest that this causes a shortage of substrate for the peptidoglycan biosynthesis enzyme MraY, causing it to be hypersensitive to competitive inhibition by tunicamycin. In conclusion, our results constitute a new molecular model for antibiotic sensitivity in *S. aureus* and a promising new route for antibiotic discovery.

The steady advance of antibiotic resistance is a significant threat to modern medicine (1, 2). *Staphylococcus aureus* is one of the most common hospital- and community-acquired opportunistic pathogens, and the fact that it has emerged in resistant forms is therefore especially concerning. The widely disseminated superbug methicillin-resistant *Staphylococcus aureus* (MRSA) has exacerbated virtually all *S. aureus* infections; ∼20% of human MRSA bacteremias are fatal (3). A multidrug resistant form known as vancomycin-intermediate *Staphylococcus aureus* (VISA) has also emerged (4, 5, 6). Vancomycin intermediate resistance confers low to moderate levels of resistance to antibiotics that target the cell wall and, while this resistance is not a serious clinical burden, VISA-like phenotypes have appeared in combination with methicillin and dalbavancin resistance, generating strains that are more problematic still (7, 8). Developing new drugs to manage resistant strains is, arguably, the most important goal in microbiology (9, 10).

Antibiotics discovered through screens of natural products and synthetic compound libraries yielded the antibiotic scaffolds in current use (11). Most of these have since succumbed to antibiotic resistance mechanisms. Chemical remodeling has generated newer compounds that bypass resistance; however, these, in turn, have also given way to new forms of resistance. This steady accumulation of resistance suggests that new approaches to antibiotic discovery are needed. Future approaches will take advantage of new knowledge about the complex molecular networks that determine antibiotic sensitivity and resistance (12).

Vancomycin and ramoplanin are two important antibiotics used to manage Gram-positive infections, including resistant forms such as MRSA. Both target components of lipid II cycle. Low to moderate level multidrug resistance to these antibiotics classified as VISA has been attributed to numerous genetic loci including two-component systems (*vraRS*, *graRS*, and *walRK*), cell wall biosynthetic enzymes (*uppS*, *fmtC*, *srtA*, and *msrR*), and even RNA polymerase (*rpoB*) (13, 14, 15). Such strains are typically also resistant to daptomycin, another drug of last resort that targets peptidoglycan biosynthesis (16). Additional phenotypic traits such as a thickened cell wall and compromised autolysis suggest that defects in cell wall homeostasis underlie this resistance (17, 18). At present however, we lack a detailed molecular description of the VISA cell.

In previous work, we isolated *S. aureus* mutants that were resistant to the antibiotics siamycin-I and actinorhodin (19, 20). These strains had mutations in the *wal* operon, which encodes a response regulator, WalR, its cognate sensor kinase, WalK, and two WalK-regulatory proteins, WalH and WalI (21, 22, 23). They constitute one of the only known two-component systems that are essential for viability in bacteria, indicating that it controls molecular processes that are vitally important. In further support of this, these genes are highly conserved in many Gram-positive bacteria including pathogens such as *Enterococcus* (YycFG), *Streptococcus* (VicKR), and even *Mycobacteria* (MtrAB) (24, 25, 26). A fascinating element in this mechanism is that, in addition to WalK, a eukaryotic-like Ser/Thr kinase, PknB, also phosphorylates WalR (27). The sites of WalR phosphorylation are distinct: WalK phosphorylates D53, and PknB phosphorylates T101 (27, 28). This is a remarkable departure from the two-component paradigm; very few response regulators are known to be phosphorylated at two sites. At present, the molecular role of phosphorylation at T101 is not well understood. WalR regulates genes involved in cell wall homeostasis. This includes several autolysin genes known for their sugar-cleaving and peptide-cleaving activity within the peptidoglycan layer permitting cell wall growth (29).

In this work, we report a detailed investigation of a *walR* point mutant that changes T101 to a methionine, eliminating the site thought to be phosphorylated by PknB. We show that this mutation confers resistance to vancomycin, ramoplanin, daptomycin, and several other antibiotics that target the cell envelope. It also has a thickened cell wall and impaired autolysis; reminiscent of clinical VISA. Surprisingly, we also find that the mutant is exquisitely sensitive to the antibiotic tunicamycin, which targets the enzymes MraY and TarO, involved in peptidoglycan and teichoic acid biosynthesis, respectively (30). This is the first indication that there is a connection between these pathways, and it suggests that in addition to its direct targets, there are indirect effects of *wal* gene mutations that make important contributions to antibiotic sensitivity and resistance. Remarkably, tunicamycin-resistant mutants selected in the *walR* mutant background reversed all the phenotypes described thus far, demonstrating a foundational molecular linkage. Using RNA-Seq, we found that the partial collapse of a pathway consisting of two autolysins and the peptidoglycan-recycling proteins, MupG, MurQ, and MurP, explains most of the antibiotic sensitivity and resistance in this mutant. With these data in hand, we have developed the first detailed molecular model for how a mutation in the *wal* operon can confer both vancomycin resistance and tunicamycin sensitivity. This in turn suggests that understanding *wal* operon mutants such as the one we described in this work will open up new opportunities for antibiotic discovery.

## Results

### Tunicamycin sensitivity in a multidrug-resistant *walR1* mutant

We selected a representative mutant from our previous work (20) encoding a T101M alteration in WalR. This strain has no other chromosome sequence changes relative to its congenic parent, *S. aureus* ATCC29213; we refer to this mutation as *walR1* (BioProject: PRJNA833295). This mutant is of particular interest because it alters the site of phosphorylation by PknB, one of the least understood aspects of this mechanism. We determined the minimal inhibitory concentration (MIC) of *walR1* and the parent strain for antibiotics that act on the five major antimicrobial targets (Table 1). The *walR1* mutant exhibited reduced sensitivity to vancomycin (two-fold), siamycin-I (four-fold), ramoplanin (four-fold), and daptomycin (two-fold), all of which target the cell wall or membrane. It also exhibited reduced sensitivity to rifampicin (two-fold), which targets RNA polymerase. In contrast, there was no change in sensitivity to the penicillin-binding protein (PBP)–targeting β-lactam ampicillin or to compounds that target DNA gyrase (ciprofloxacin), the folate pathway (trimethoprim), and the ribosome (kanamycin). Aside from rifampicin, the strain’s resistance profile was restricted to compounds that act *via* lipid II pathway (vancomycin, siamycin-I, and ramoplanin) and the cell membrane (daptomycin).

**Table 1:** MICs of each strain against a panel of antibiotics

MIC (μg/ml)			
Drug	Wildtype	*walR1*	*walR1–walK*^tun^
Tunicamycin	8	0.125	8
Siamycin-I	16	64	16
Ramoplanin	0.5	2	1
Vancomycin	1	2	1
Mecillinam	64	32	64
Cefaclor	1	0.5	1
Cefoxitin	2	1	2
Oxacillin	0.125	0.125	0.125
Ceftriaxone	2	2	2
Daptomycin	1	2	1
Ciprofloxacin	0.25	0.25	0.25
Kanamycin	8	8	8
Rifampicin	0.0078	0.0156	0.0078
Trimethoprim	2	2	2

**Figure 1:**
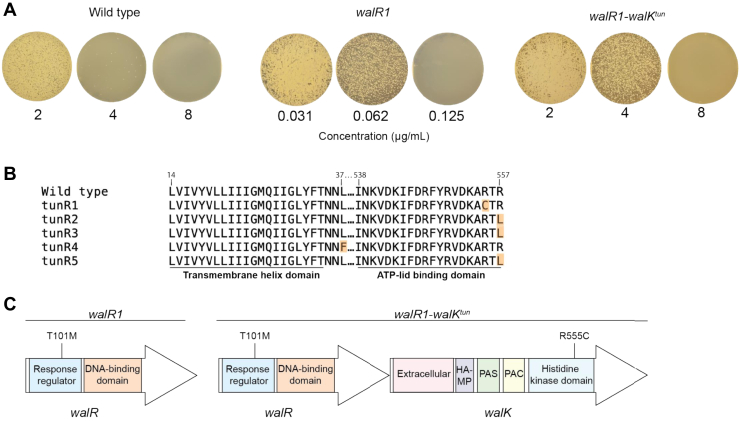
**Single-point mutations that confer and reverses tunicamycin sensitivity.***A*, bactericidal concentrations of tunicamycin against wildtype *Staphylococcus aureus* ATCC29213, *walR1*, and *walR1–walK*^*tun*^, plated on TSB agar. *B*, multiple sequence alignment of tunicamycin-resistant (tun^R^) mutants. Mutations were mapped to the histidine kinase domain of WalK: four of five mutations were found in the ATP-lid binding domain and one of five was mapped outside a transmembrane helix domain. *C*, point mutations observed in *walR1* and *walR1–walK*^*tun*^. HAMP, histidine kinase, adenylate cyclase, methyl accepting protein and phosphatase; PAC, C-terminal motif of PAS; PAS, Per-Arnt-Sim; TSB, tryptic soy broth.

To our considerable surprise, the *walR1* mutant also exhibited a 64-fold increase in susceptibility to tunicamycin with a change in MIC from 8 to 0.125 μg/ml. The effect was bactericidal (Fig. 1*A*). Tunicamycin targets the MraY and TarO proteins, involved in the early steps of peptidoglycan and teichoic acid biosynthesis, respectively (30). We therefore expanded this analysis to more selective cell wall–targeting β-lactams (31). We observed no change in sensitivity to oxacillin (PBP2 and PBP3 specific) and ceftriaxone (PBP2 specific) and a twofold increase in sensitivity to mecillinam and cefaclor (PBP3 specific), and cefoxitin (PBP4 specific) (Table 1). The dramatic 64-fold increase in sensitivity to tunicamycin was specific to that antibiotic. This is unusual—we know of no other example where resistance to one set of antibiotics comes at the cost of such extreme sensitivity to another.

To better understand this phenomenon, we isolated six nonsibling tunicamycin-resistant mutants (tun^R1–6^) in the *walR1* background. All six mutants displayed tunicamycin sensitivity comparable to the wildtype parent strain (MIC = 8 μg/ml). Upon whole-genome sequencing of these mutants, we found that five had sequence changes in the *walK* gene. Four mapped to the “lid” motif in the ATP-binding pocket and one at the cytoplasmic face of the second transmembrane helix of WalK (Fig. 1*B*). The sixth suppressor mutation was found within the *clpP* gene (C478T nucleotide change), which encodes a highly conserved ATP-dependent protease (32), and is predicted to cause a ClpP truncation (Table S3). The T101M sequence change of the *walR1* allele was unchanged in all six strains. The fact that five of the six mutations in these strains mapped within *walK* underscores the intimate connection between the *wal* operon and antibiotic activity in *S. aureus.*

To continue this investigation, we selected a mutant that has a single sequence change relative to the *walR1* mutant, namely a R555C polymorphism in the WalK ATP-binding pocket, for further characterization (Fig. 1*C*). This mutant is henceforth referred to as *walK*^*tun*^. Remarkably, the *walR1–walK*^*tun*^ double mutant exhibited normal sensitivity to vancomycin, siamycin-I, daptomycin, mecillinam, cefaclor and cefoxitin, rifampicin, as well as tunicamycin (Table 2). This suggests that the mutations found in the *walR1* and *walK*^*tun*^ strains are toggling the physiological state of the cell between two states. It also underscores the fact that *walRK-*mediated resistance to vancomycin and other antibiotics comes at the cost of sensitivity to tunicamycin: these phenotypes change in lock step fashion.

**Table 2:** MICs of *walR1* complemented with an empty vector (+EV) control or the *mur* operon (+p*GQP*)

MIC (μg/ml)		
Drug	+EV	+p*GQP*
Tunicamycin	0.25	> 8
Siamycin-I	64	16
Ramoplanin	1	1
Vancomycin	4	2
Cefaclor	1	1
Cefoxitin	2	4
Daptomycin	16	8
Ciprofloxacin	0.5	0.5
Kanamycin	8	8
Rifampicin	0.0078	0.0039

### Alterations in cell wall, septation, and autolysis in the *walR1* mutant

**Figure 2:**
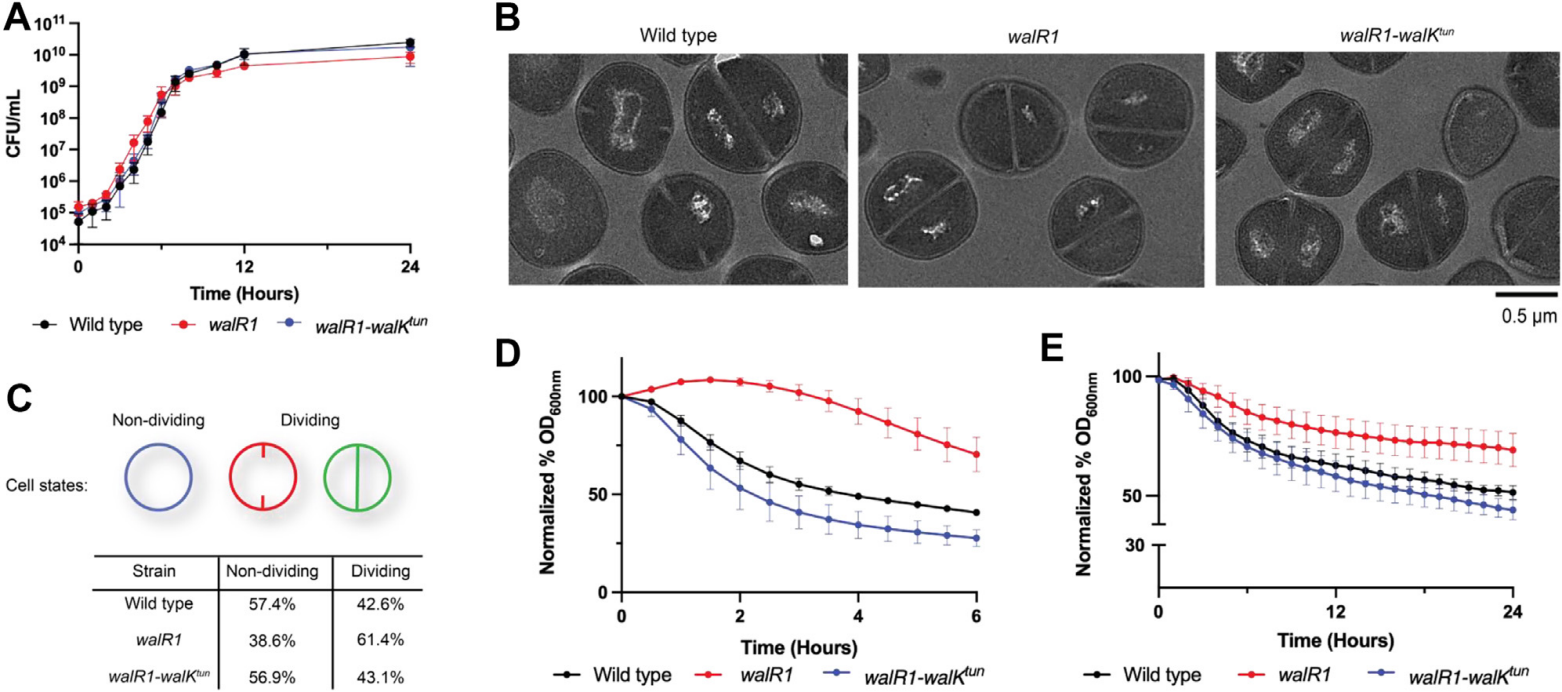
**Characterization of the cell wall and autolytic activity in *walR1*.***A*, growth curve of wildtype, *walR1*, and *walR1–walK*^*tun*^ measured by colony-forming unit (CFU)/ml over 24 h; n = 3, ±SD. *B*, transmission electron micrographs of each strain during log-phase growth. Cell wall thicknesses were quantified over 350 cells: wildtype (34.1 nm), *walR1* (53.9 nm), and *walR1–walK*^*tun*^ (30.7 nm). *C*, percentage of cells in either a dividing or a nondividing state based on the 350 cells from transmission electron microscopy. *D*, autolytic activity of each strain in the presence of 0.05% (v/v) Triton X-100 measured by the change in turbidity at an absorbance at 600 nm over 6 h; n = 3, ±SD. *E*, crude autolysin activity from each strain on wildtype peptidoglycan as a substrate measured by the change in turbidity at an absorbance at 600 nm over 24 h; n = 3, ±SD.

To determine whether the effects of *walR1* and *walR1–walK*^*tun*^ on antibiotic responsiveness extended to other phenotypes, we investigated changes in cell structure and growth. We found that the growth curves of the wildtype, *walR1*, and *walR1–walK*^*tun*^ strains were similar with generation times of 23.4, 23.1, and 21.5 min, respectively (Fig. 2*A*). We visualized log phase cells of the three strains using transmission electron microscopy to compare their cell wall thickness and cell division status. Consistent with the literature for wildtype *S. aureus* and *wal* mutants (18), wildtype cells exhibited a cell wall thickness of 34.1 ± 1.4 nm. In contrast, the *walR1* mutant exhibited a cell wall thickness of 53.9 ± 0.9 nm. The cell wall of the *walR1–walK*^*tun*^ strain was almost identical to that of the wildtype parent at 30.7 ± 0.8 nm (Fig. 2*B*). Despite this difference in cell wall thickness, the cell wall composition was similar between wildtype and *walR1* bacteria (Fig. S1 and Table S4). We compared the septation state of 350 cells for each of the wildtype, *walR1*, and *walR1–walK*^*tun*^ strains (Fig. 2*C*). This analysis revealed that 42.6% of wildtype cells had clear division septa or preseptal invagination with no observable evidence of a division septum in the other 57.4%. In contrast, 61.4% of the *walR1* mutant cells showed division septa and 38.6% did not. The *walR1–walK*^*tun*^ cells resembled the wildtype with 43.1% of cells displaying a division septum and 56.9% exhibiting no division septum. The apparent differences in the distribution of cells having septa suggest that *walR1* bacteria might be slightly impaired in transit through septation and on to the separation of daughter cells, though apparently, this defect does not alter the growth rate. Most importantly, the effects of the *walR1* mutation on cell wall thickness and septation were reversed in the *walR1–walK*^*tun*^ mutant.

It is known that *wal* operon mutations alter the expression of autolysin genes (29). We therefore compared the autolysis profile of each strain. To this end, we resuspended exponential phase cells in 0.05% (v/v) Triton X-100 and measured the absorbance at 600 nm of each suspension as a function of time. Consistent with published reports, wildtype *S. aureus* underwent efficient autolysis: culture turbidity decreased by 59% over the 6 h time course. In contrast, the *walR1* mutant displayed reduced lysis in the presence of the detergent as culture turbidity decreased by only 30% over 6 h with readily distinguishable kinetics. The *walR1–walK*^*tun*^ mutant displayed a more normal autolytic profile with a 72% reduction in culture turbidity indicating that the R555C substitution in WalK again suppressed the effects of the T101M mutation in WalR (Fig. 2*D*).

To further analyze autolysis, we adapted an established biochemical assay to monitor autolysin activity *in vitro* (33). We derived protein extracts from each strain and assessed their capacity to degrade peptidoglycan isolated from the wildtype parent over 24 h using absorbance measured at 600 nm to monitor progression. The wildtype *S. aureus* extract caused a 49% reduction in turbidity over the time course, whereas the *walR1* mutant extract caused only a 30% reduction. As in previous experiments, the *walR1–walK*^*tun*^ strain exhibited restored peptidoglycan degradation with a 44% reduction in turbidity (Fig. 2*E*).

These data demonstrate that the *walR1* mutation reduces autolysin activity and that the *walR1–walK*^*tun*^ mutation restores it. Again, all phenotypes change in lock step, consistent with an integrated cause-and-effect relationship.

### The *walR1* phenotype is due to a defect in cell wall recycling

**Figure 3:**
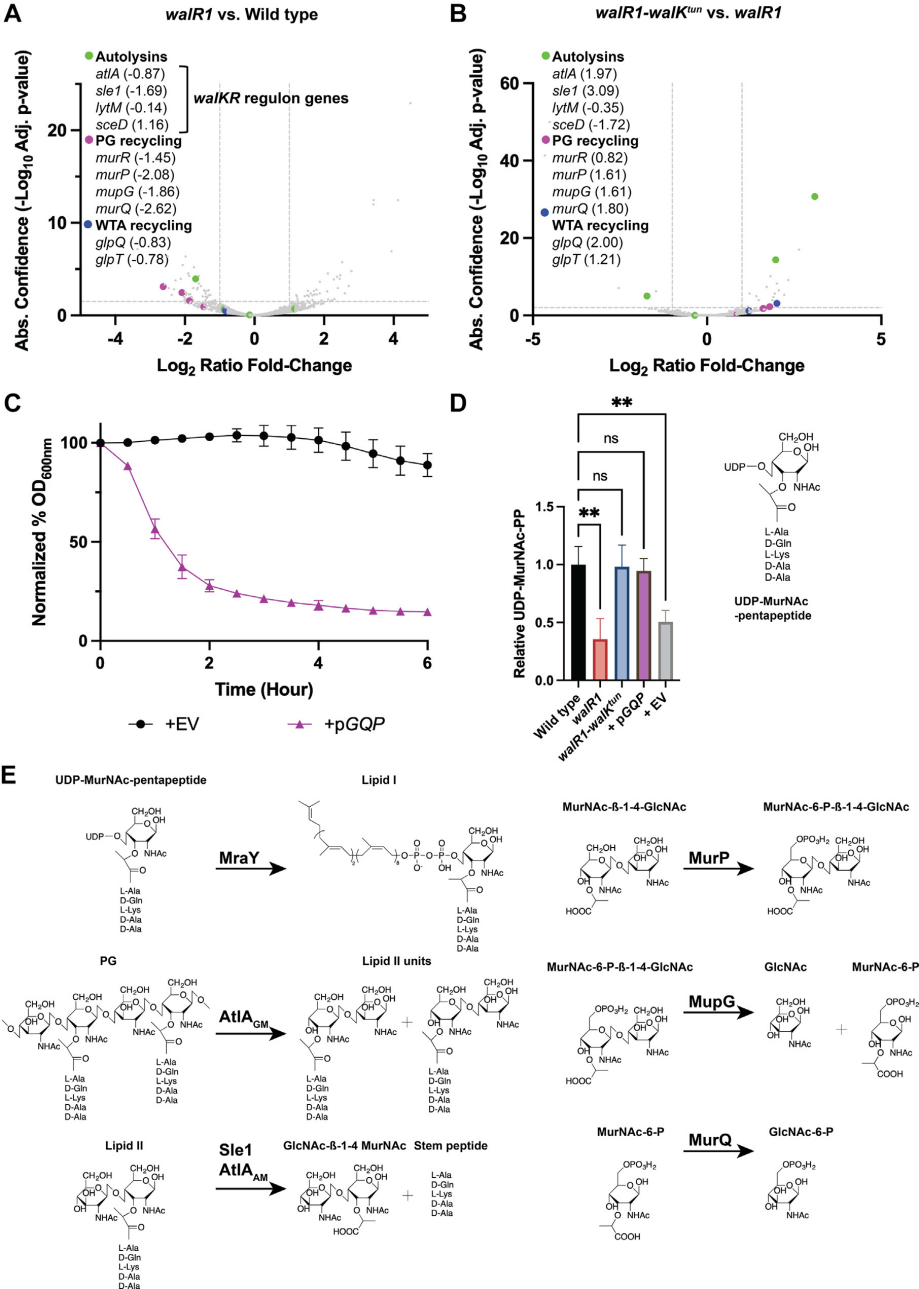
***walR1* changes are caused by a defect in peptidoglycan recycling.***A*, RNA-Seq analysis of *walR1* relative to wildtype cells in logarithmic phase. Data points above the *dashed lines* along the *y*-axis represent *p* ≤ 0.01. Data points outside the *dashed lines* along the *x*-axis represent fold changes ≥2. Results are from three independent biological replicates. Genes of interest are highlighted in the graph, and log_2_ ratio fold changes are indicated in parentheses. *B*, RNA-Seq analysis of *walR1–walK*^*tun*^ relative to *walR1* in logarithmic phase. *C*, autolytic activity of *walR1* complemented with either an empty vector control (+EV) or the *mur* operon, +p*GQP* (*mupG murQ murP*), in the presence of 0.05% Triton X-100 measured by the change in an absorbance at 600 nm over 6 h; n = 3, ±SD. *D*, relative quantification of UDP-MurNAc-pentapeptide (PP) by LC–MS/MS. The cytoplasmic UDP-MurNAc-PP was extracted from log phase cells in the presence of 10× MIC vancomycin to block peptidoglycan biosynthesis, and the *m/z* fragment ion 746.35 (MurNAc-PP) was quantified using TargetLynx Software (Waters). Results are from three independent biological replicates. Statistical significance was assessed by one-way ANOVA using Tukey’s *t* test; ∗∗ denotes *p* < 0.01, ns denotes not significant. *E*, biochemical reactions of enzymes involved in peptidoglycan biosynthesis (MraY), hydrolysis (AtlA and Sle1), and recycling (MurP, MupG, and MurQ). AM, amidase domain; GM, glucosaminidase domain; MIC, minimal inhibitory concentration; MurNAc, *N*-acetyl muramic acid; P, phosphate; PG, peptidoglycan.

A plausible hypothesis is that the myriad effects of the *walR1* and *walR1–walK*^*tun*^ mutations are caused by altered expression of the WalR regulon. Given that *walKR* is essential for viability, we surmised that the *walR1* mutation is partially defective in WalRK-mediated signal transduction and that *walK*^*tun*^ might reverse its effects, restoring a more normal transcriptome. To determine how WalR regulated gene expression is affected in the *walR1* and the *walR1–walK*^*tun*^ strains, we carried out an RNA-Seq analysis (transcriptomic data are accessible through Gene Expression Omnibus Series accession number GSE211745). We first compared transcript abundance in wildtype *S. aureus* with the *walR1* mutant strain. Consistent with previous work, we observed reduced transcript abundance in at least two known WalR-target genes, *atlA* and *sle1*, both of which encode autolysins (29). AtlA is a bifunctional glucosaminidase (GM) and amidase (AM). AtlA_GM_ cleaves the β-1,4-glycosidic bond between GlcNAc and MurNAc, whereas AtlA_AM_ removes the stem peptide from MurNAc. Sle1 is also an *N*-acetylmuramyl-l-alanine amidase and acts on the same substrate as AtlA_AM_ (34) (Fig. 3*E*). Transcript abundance for the *lytM-*encoded autolysin was unchanged and, paradoxically, that of *sceD* appeared to be increased (Fig. 3*A*). A reduction in *atlA* and *sle1* transcript abundance is consistent with reduced autolysis in the *walR1* mutant.

In addition however, we observed reduced transcript abundance for the monocistronic genes *glpQ* and *glpT*, involved in glycerophosphate recycling (35), as well as the *mupG murQ murP murR* operon, which encodes a peptidoglycan-recycling pathway (36). The products of this operon include a transmembrane permease (MurP), which imports GlcNAc–MurNAc, the products of autolysis, into the cytoplasm and phosphorylates it. MupG hydrolyzes the glycosidic bond and forms GlcNAc and MurNAc-6-phosphate as end products. MurQ then converts MurNAc-6-phosphate into GlcNAc-6-phosphate (36, 37) (Fig. 3*D*). MurR is a putative repressor of the *mupG* promoter that presumably regulates the expression of all four genes. The entire operon was downregulated in *walR1*. This is the first evidence of a possible link between the expression of these cell wall recycling genes and WalR.

Based on the ability of the *walK*^*tun*^ mutation to toggle the *walR1* mutant back to an approximately wildtype phenotype, we hypothesized that the most important genes that were downregulated in *walR1* would be upregulated in *walR1–walK*^*tun*^. Therefore, we performed a differential expression analysis comparing our *walR1–walK*^*tun*^ double mutant relative to the parental *walR1* mutant. This analysis revealed that transcript abundance for autolysins, *atlA* and *sle1*, was upregulated, whereas *sceD* was downregulated and *lytM* remained unchanged. The genes involved in glycerophosphate and peptidoglycan recycling were also upregulated (Fig. 3*B*). This analysis further corroborates the suppressor effect of the WalK R555C mutation of the *walR1–walK*^*tun*^ mutant on the tunicamycin-sensitive *walR1* strain. More importantly, it also suggests that one or both these cell wall recycling pathways might underpin the effects of the *walR1* mutation on tunicamycin sensitivity.

We were particularly interested in the *mupG* operon because the product of this recycling pathway, GlcNAc-6-phosphate, is used to generate UDP-MurNAc-pentapeptide (PP), the substrate of MraY, one of two tunicamycin target proteins. To test the hypothesis that reduced expression of these genes might be related to the antibiotic resistance and sensitivity in the *walR1* mutant, we created the plasmid p*GQP* in which the *mupG*, *murQ*, and *murP* genes were placed under the control of an anhydrotetracycline-inducible promoter (38). We introduced p*GQP* into the *walR1* mutant and assessed its effect on antibiotic resistance in comparison with the *walR1* bacteria carrying the empty vector. Remarkably, p*GQP* restored the *walR1* mutant to a nearly wildtype phenotype. Tunicamycin sensitivity was reduced from an MIC of 0.125 to 8 μg/ml; identical to that of the wildtype strain and the *walR1–walK*^*tun*^ strain. Moreover, sensitivity to siamycin-I, vancomycin, daptomycin, cefoxitin, and rifampicin was also restored to wildtype levels. These effects were specific: p*GQP* had no effect on MICs for kanamycin or ciprofloxacin (Table 2). Sensitivity to ramoplanin and cefaclor was both unaffected.

We also assessed the effect of *mupG*, *murQ*, and *murP* expression on autolysis. As expected, *walR1* bacteria containing the empty vector exhibited the impaired autolysis consistent with the effects described previously. In contrast, *walR1* bacteria carrying plasmid p*GQP* displayed autolysis, akin to wildtype with an endpoint absorbance that was reduced by ∼86% (Fig. 3*C*).

Finally, we hypothesized that a peptidoglycan recycling defect should cause a depletion in the intracellular pool of peptidoglycan precursors such as the UDP-MurNAc-PP, the cytoplasmic substrate that is attached to the lipid carrier undecaprenyl phosphate by MraY (Fig. 3*E*). To assess this, we employed a previously developed assay in which vancomycin is used to halt peptidoglycan crosslinking outside the cell. This creates a “log-jam” effect resulting in the accumulation of intracellular peptidoglycan intermediates (39). We then quantified the amounts of UDP-MurNAc-PP in each cell. Comparison of UDP-MurNAc-PP in the three strains demonstrated that this penultimate cytoplasmic intermediate was specifically reduced in the *walR1* mutant by ∼75% relative to wildtype. The suppressor mutation *walK*^*tun*^ restored levels of intracellular UDP-MurNAc-PP to an approximately wildtype level. Most importantly, the p*GQP* plasmid also restored UDP-MurNAc-PP to near wildtype levels of 94% (Fig. 3*D*). This is particularly remarkable because it did so without manipulation of the autolysin genes themselves.

In sum, we find that the *walR1* mutation causes the partial collapse of a pathway consisting of the extracellular autolysins AtlA and Sle1 and the peptidoglycan-recycling pathway encoded by *mupG murP* and *murQ*. In spite of the fact that the *mupG* promoter is not a known WalR target, the role of this peptidoglycan-recycling pathway is so important that its restored expression is sufficient to correct most of the effects of the *walR1* mutation.

## Discussion

We have shown that the T101M mutation in the *walR1* mutant confers resistance to important experimental and clinical antibiotics as well as reduced autolysis and a thickened cell wall. We find that the mutant has an extraordinary sensitivity to tunicamycin. This is especially important because a new chemical vulnerability in an otherwise multidrug-resistant strain suggests the possibility of new avenues for managing antibiotic-resistant infections. The phenotypic effects of *walR1* were associated with reduced transcript levels for two autolysin-encoding genes (as expected based on the known WalR regulon) as well as genes that encode the cell wall recycling pathway. This includes most notably the *mupG* operon, involved in peptidoglycan recycling. Metabolomic analysis supported this: we observed reduced intracellular levels of UDP-MurNAc-PP, a downstream product of the MupG peptidoglycan-recycling pathway. All these effects could be reversed by the *walK*^*tun*^ second site suppressor mutation in WalK (R555C) or by restoring the expression of *mupG murQ* and *murP* in *walR1* bacteria. This work demonstrates a critical mechanistic role for peptidoglycan recycling, in concert with the expected role of the autolysins, in the *walR1* phenotype. This is the first demonstration of a direct link between the *wal* mutant phenotype and these cell wall recycling genes.

**Figure 4:**
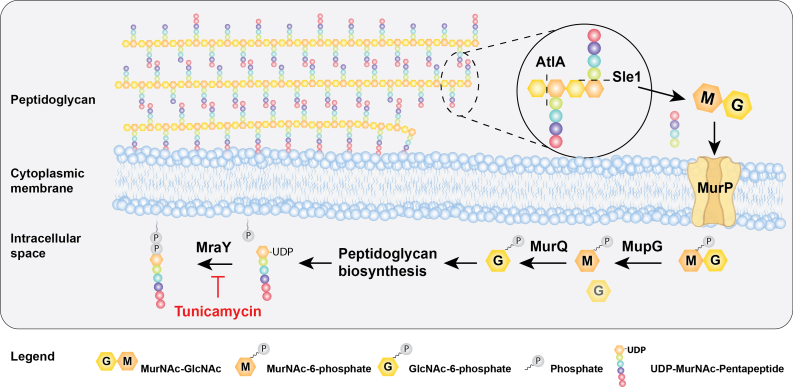
**Peptidoglycan recycling and antibiotic sensitivity and resistance.** The enzymes AtlA and Sle1 cut residues of GlcNAc–MurNAc from the cell wall. These are imported through the MurP permease. These are then converted to GlcNAc-6 phosphate by MupG and MurQ. GlcNAc-6 phosphate serves as a precursor for the MraY substrate. By downregulating this pathway, the *walR1* mutation causes increased accumulation of peptidoglycan, thus conferring resistance to many cell wall active antibiotics. At the same time, reduced substrate availability for MraY sensitizes the cell to tunicamycin. (Portions of the figure are created with BioRender.com). MurNAc, *N*-acetyl muramic acid.

These data support a new model for resistance and sensitivity to cell wall-active antibiotics in *S. aureus* (Fig. 4). According to this model, AtlA and Sle1 act on the peptidoglycan to liberate the disaccharide, GlcNAc–MurNAc. This molecule is known to be imported through the MurP permease and acted on by the enzymes MupG and MurQ to generate GlcnNAc-6-phosphate. GlcNAc-6-phosphate is used to generate UDP-MurNAc-PP, the substrate of MraY and UDP-*N*-acetylglucosamine, the substrate of TarO (36, 37, 40). Therefore, by reducing the expression and efficiency of this pathway, the mutation in *walR1* bacteria reduces the substrate availability for MraY and TarO and this, in turn, likely sensitizes those enzymes to inhibition by tunicamycin. Tunicamycin is a competitive inhibitor and so, less antibiotic would be required to compete with the substrate. The resistance of the *walR1* mutant to vancomycin, siamycin-I, ramoplanin, daptomycin, and nisin is likely to be the result of the increased bulk of cell wall in the *walR1* strain. We detected no biochemical difference in the peptidoglycan of the mutant strain aside from the fact that there was more of it (Fig. S1).

The mechanism by which the expression of the *mupG* operon is reduced in the *walR1* mutant is unknown at this time. There is no reason to think that the *mupG* promoter is a hitherto unknown member of the WalR regulon because the DNA sequence recognized by WalR, 5′-TGT(A/T)A(A/T/C)-N_5_-TGT(A/T)A(A/T/C)-3′, is absent from its promoter. A more likely explanation involves *murR*. The MurR protein is encoded by the fourth gene in the *mupG* operon, and its *Escherichia coli* ortholog was previously shown to repress the cognate operon (41). MurR consists of an N-terminal DNA-binding domain and C-terminal sugar phosphate-binding domain. It may be that this C-terminal domain binds GlcNAc-6-phosphate liberated by the autolysins and imported by MurP, resulting in allosteric derepression of the *mupG* promoter. In this scenario, the normal expression level of these genes would be determined by the activation of the autolysin genes by phosphorylated WalR and the derepression of the *mupG* promoter through the interaction of the sugars generated by the autolysins with the C-terminal domain in MurR.

The restoration of autolysis by heterologous expression of the *mupG* operon is likely to be independent of autolysin gene expression. In the *walR1* mutant, autolysin expression and activity were reduced to a lower level. We suggest that this reduced expression level is sufficient to support nearly normal production of UDP-MurNAc-PP as long as the *mupG*, *murQ*, and *murP* genes are expressed. Induced MurP expression might relieve product inhibition of the remaining autolysin proteins by drawing away their reaction products through active import.

The *walR1* mutation, T101M, is interesting in that it is similar to previously reported mutations (T101S and T101A) found in clinical VISA strains (14). The T101 residue is believed to be targeted for phosphorylation by the Ser/Thr kinase PknB (27) (also known as Stk), and defects in *pknB* have previously been linked to tunicamycin sensitivity (42). This suggests that phosphorylation of this residue is important for full activity of the WalR protein. This could indicate a role for T101 phosphorylation in DNA binding or WalR stability. Regardless, we suggest that the *walK*^*tun*^ mutations (four of which fall in the regulatory lid of the ATP-binding site in the kinase domain) create a more active WalK kinase and that this bypasses the need for phosphorylation by PknB.

We note that the links we have described between WalR and cell wall recycling are novel. The idea that reduced cell wall recycling sensitizes one or more intracellular enzymes to chemical inhibition suggests new avenues for antibiotic discovery. New compounds that specifically impair the growth of mutants like *walR1* might well act *via* these sensitized enzymes.

## Experimental procedures

### General methods and bacterial strains

Chemicals used in this study were purchased from Sigma–Aldrich unless stated otherwise. Tunicamycin was purchased from Cayman Chemical. All *S. aureus* and *E. coli* strains were grown in tryptic soy broth (TSB) or LB, respectively, at 37 °C with shaking at 220 RPM or on TSB/LB agar plates in a stationary 37 °C incubator unless otherwise stated. All strains used in this study are listed in Table S1. All experiments were performed in biological triplicates with at least two technical replicates unless stated otherwise.

### Microtiter broth dilution assays

Cultures were grown overnight and subcultured the next day in 1:1000 dilution in fresh media. Cultures for complementation with pRMC2 were grown in 12 μg/ml chloramphenicol, and no anydrotetracycline was required for induction. Bacterial cells were incubated and grown until exponential phase (absorbance at 600 nm = 0.4) and then subcultured in 1:10,000 dilution in fresh media. About 198 μl of culture was aliquoted into each well in a 96-well plate and inoculated with 2 μl of drug. About 50 μg/ml of Ca^2+^ was added into TSB media for daptomycin only. A vehicle control and blank media control were included. Plates were incubated overnight for 18 to 20 h, and the turbidity of wells was read with an absorbance at 600 nm using the EPOCH plate reader (BioTek).

### Whole genome sequencing of resistant mutants

Resistant mutants were raised against tunicamycin by serial passaging individual nonsibling colonies of *walR1* in a twofold series of increasing concentration of tunicamycin. From day 1, strains were inoculated with 1/2× MIC and then subcultured each day in 1:1000 dilution with twofold increases of drug up to 32× MIC. Genomic DNA from each mutant was isolated using the DNeasy Blood & Tissue Kit (Qiagen). To lyse open *S. aureus*, cultures were incubated with lysostaphin (200 μg/ml in 20 mM Tris–HCl [pH 7.5] and 10 mM EDTA).

Sample libraries were prepared with the Illumina Nextera XT kit and subjected to whole genome sequencing with v2 chemistry, paired-end reads (2 × 150 bp) on an Illumina MiSeq platform. Sequencing and library preparation was performed at the Centre for the Analysis of Genome Evolution and Function at the University of Toronto. Raw reads were processed, assembled, and mapped using Geneious (version 9.1) to *S. aureus* ATCC29213 (GenBank accession: GCA_001889295.1) with coverage cutoffs of 100× and cutoff frequency of at least 95% for detection of mutations.

### Growth curve and colony-forming unit assays

Starter cultures were grown overnight, subcultured the next day in 1:10,000 dilution in fresh media, and incubated at 37 °C with shaking for 24 h. At each timepoint, the absorbance at 600 nm was measured with an EPOCH plate reader (BioTek). An aliquot of sample was also taken and serial diluted in a 10-fold dilution series. Serial dilutions were plated on TSB agar plates and incubated in a stationary incubator for 16 h at 37 °C. Colonies were enumerated the next day.

### Cloning of pRMC2 constructs

All primers used in this study are listed in Table S2. *mupG*, *murQ*, and *murP* were PCR amplified from wildtype ATCC29213 genomic DNA to include KpnI and EcoRI cut sites, a 6× His tag, and an RBS as one single amplicon. PCR amplicons and the plasmid pRMC2 were digested with KpnI and EcoRI and then ligated and transformed into *E. coli* Stellar competent cells on LB ampicillin (100 μg/ml). Plasmids were passaged through *S. aureus* RN4220 before electroporating into the final strain and selected for on TSB chloramphenicol (12 μg/ml). Constructs were confirmed by miniprepping the plasmid for digestion of the insert and by sequencing.

### Transmission electron microscopy

Cultures were grown overnight and subcultured the next day in 1:1000 dilution in fresh media. Bacterial cells were grown until exponential phase (absorbance at 600 nm = 0.4) and harvested by centrifugation at 2500*g* for 5 min. Samples were fixed with 1% osmium tetroxide, stained with 1% uranyl acetate, dehydrated using a gradient series of ethanol (30%, 50%, 70%, and 95%) twice, and finally with 100% ethanol. Between each step, samples were washed with double-distilled water (ddH_2_O). Samples were then washed with propylene oxide and infiltrated with Spurr’s resin and propylene oxide mixtures as follows: 50% resin for 30 min, 75% resin for 1 h, and 100% resin overnight at room temperature with rotation. Pellets were placed in polyethylene BEEM capsules with fresh resin and left to polymerize in the oven at 60 °C for 48 h. Samples are sectioned on a Reichert Ultracut E microtome to 100 mm thickness and collected on 300 mesh copper grids. Sections were positively stained in saturated uranyl acetate for 15 min followed by Reynold’s lead citrate for 15 min. Micrographs were acquired using a TALOS L120C electron microscope at an accelerating voltage of 120 kV (Thermo Fisher Scientific). Images were processed and analyzed in ImageJ (U.S. National Institutes of Health).

### *In vivo* autolysis activity assays

An overnight culture of bacteria was grown and subcultured the next day to obtain an absorbance of 0.05 at 600 nm. Once cells reached early log phase growth (absorbance at 600 nm = 0.4), cells were chilled on ice immediately and harvested by centrifugation at 10,000*g* for 15 min at 4 °C. Pellets were washed twice with cold sterile ddH_2_O. Pellets were collected and resuspended in 50 mM Tris–HCl (pH 7.4) + 0.05% (v/v) Triton X-100 to an absorbance of 1.0 at 600 nm. Samples were aliquoted into a clear sterile 96-well plate in triplicate, and absorbance at 600 nm was measured every 30 min for 6 h at 37 °C with shaking in a Synergy plate reader (BioTek).

### *In vitro* autolysis activity assays

Crude autolysin extracts were prepared as described with the following additions (33). The pellet after 4% SDS treatment was saved and frozen at −20 °C for whole-cell substrate preparation. Pellets were thawed at room temperature and boiled in 1:50 volume (of original culture) of 8% SDS for 1 h on a heating block. Samples were cooled to room temperature after, and cells were centrifuged for 3 min at 13,100*g*. Next, samples were washed with sterile ddH_2_O five times to remove all trace amounts of SDS. Samples were then incubated in 1:25 volume (of original culture) of buffer (30 mM Tris–HCl [pH 8.0]) with 200 μg/ml proteinase K at 50 °C for 1.5 h. After digestion, samples were centrifuged for 10 min and boiled again in 1:50 volume of 8% SDS for 1 h in a heating block. Repeated washing with sterile ddH_2_O was performed five times to remove all residual SDS. On the last wash, supernatant was removed, the pellet was frozen in liquid nitrogen, and lyophilized overnight (Labconco).

The dried substrate was resuspended in 50 mM K_2_HPO_4_/KH_2_PO_4_ buffer (pH 7.3) to an absorbance of 0.6 at 600 nm. Total protein extracts (containing the autolysins) were added to the cell wall substrate suspension to a final concentration of 15 μg/ml and aliquoted into a clear sterile 96-well plate. The plate was then incubated at 37 °C with shaking in the Synergy plate reader for 24 h, and absorbance at 600 nm was measured every hour.

### RNA extraction

Single colonies of strain(s) of interest were inoculated overnight at 37 °C. Strains were subcultured the next day in 1:10,000 dilution in 25 ml of fresh media, and aliquots were taken at early log (3.5 h, absorbance at 600 nm = ∼0.2), midlog (6 h, absorbance at 600 nm = ∼0.9), and stationary (12 h, absorbance at 600 nm = ∼1.5.) of incubation (time points were chosen based on growth curves of each strain). Samples were pelleted, washed with RNAse-free water twice, and stored at −80 °C until further processing. RNA was isolated from samples using the Qiagen RNeasy Mini Kit as instructed (43). Briefly, pellets were thawed and resuspended in 100 μl TE buffer (pH 8.0) with <106 μm beads. Samples were bead beated for 60 s and placed back on ice. About 650 μl of buffer RLT (Qiagen) containing β-mercaptoethanol was added to each sample and bead beated for an additional 60 s. Samples were centrifuged at 4 °C at 13,100*g* for 1 min. Supernatants were collected and mixed with 900 μl of 100% ethanol. Further processing of RNA was continued as per Qiagen’s protocol. Isolated RNA was treated with RNAse-free DNAse (Qiagen). Concentrations and purity were determined by Nanodrop and agarose gel.

### RNA-Seq and RNA analysis

RNA samples were processed by the Microbial Genome Sequencing Center in Pittsburgh, PA. Ribosomal RNA was depleted using RiboZero Plus kit (Illumina), and complementary DNA libraries were generated with Stranded Total RNA Prep Ligation kit (Illumina). Sequencing was performed using a NextSeq2000 to produce 2 × 50 bp reads. Adaptors were trimmed, and sequences were demultiplexed with the bcl2fastq tool. Sequence reads were mapped to the reference *S. aureus* N315 genome (GCF_000009645.1) using Geneious Prime 2021.2.1 (Biomatters). Expression analysis and comparisons were performed using DeSeq2 (Bioconductor) (44). All transcriptomic data are accessible through Gene Expression Omnibus Series accession number GSE211745.

### UDP-MurNAc-PP accumulation assay

The cytoplasmic precursor of peptidoglycan, UDP-MurNAc-PP, was extracted and analyzed as described (39). In brief, cells were subcultured in 1:1000 dilution with antibiotic as needed. Cells were grown to an absorbance of 0.4 at 600 nm, where 130 μg/ml of chloramphenicol was added and incubated for another 15 min to inhibit protein synthesis. Vancomycin was then added at 10× MIC (10 μg/ml) to samples and incubated in the shaker for an additional hour to halt peptidoglycan biosynthesis. Samples were then centrifuged for 5 min at 13,100*g* at room temperature. Pellets were resuspended in 30 μl of ddH_2_O and boiled for 15 min on a heat block. The supernatant was collected by centrifugation at 13,100*g* for 5 min.

### Quantification of UDP-MurNAc-PP by LC–MS/MS

A 1:100 dilution of the supernatant from the accumulation assay was prepared by diluting into 1:1 LC–MS grade H_2_O:MeCN + 0.1% formic acid. Quantification of the precursor was performed by LC–MS/MS using a Waters Alliance I-Class coupled to a Xevo G2-S QToF. About 10 μl of each sample was injected into an Acquity UPLC BEH C18 column (2.1 × 50 mm, 1.7 μm) at 40 °C. A flow rate of 0.1 ml/min was applied. Solvents used were (A) H_2_O + 0.1% formic acid and (B) MeCN + 0.1% formic acid with the following protocol for separation: 5% B (0–5 min), 5 to 95% B (5–7 min), 95 to 5% B (7–8 min), and 5% B (8–10 min). UDP-MurNAc-PP was identified as an ion of [M + H]^+^ 1150.35, and quantification was performed on the fragment ion *m/z* 746.35 using TargetLynx software (Waters).

### Graphical and statistical analysis

Statistical analyses were performed using GraphPad Prism software, version 9.3.1 (GraphPad Software, Inc). Statistical significance was calculated using ANOVA and Student’s *t* tests with SD as appropriate.

## Data availability

All data are available within the article and supporting information. All material and correspondence should be directed to justin.nodwell@utoronto.ca.

## Supporting information

This article contains supporting information (38).

## Conflict of interest

The authors declare that they have no conflicts of interest with the contents of this article.
