# Supplementary material for: A defect in cell wall recycling confers antibiotic resistance and sensitivity in Staphylococcus aureus
Source: J Biol Chem. 2022 Sep 9;298(10):102473. doi: 10.1016/j.jbc.2022.102473 (PMC9547203; doi:10.1016/j.jbc.2022.102473)
Supplement: Supplementary Data 1 [file mmc1.pdf]

Supplemental Table 1 – Bacterial strains and plasmids used in this study

Strain	Description	Source
S. aureus ATCC 29213	*Staphylococcus aureus*	ATCC
S. aureus RN4220	Restriction deficient *S. aureus* cloning strain; mutation in *sau1* and *hsdR*; *mec-*, *rsbU-* and *agr-*	Lab stock
walR1	*S. aureus* ATCC29213 with $\mathrm{WalR_{T101M}}$ chromosome mutation	Lab stock
walR1-walK^{tun}	*S. aureus* ATCC29213 with $\mathrm{WalR_{T101M}}$ and $\mathrm{WalK_{R555C}}$ chromosome mutation	This study
pRMC2	Anhydro-tetracycline inducible plasmid for *S. aureus*	Corrigan *et al.*[1]
S. aureus RN4220::pRMC2 (EV)	*S. aureus* RN4220 carrying empty vector pRMC2	This study
S. aureus RN4220::p*GQP*	*S. aureus* RN4220 carrying pRMC2 with *mupG-murQR* operon from *S. aureus* ATCC29213	This study
walR1::pRMC2 (EV)	*walR*$_{\mathrm{T101M}}$ harbouring pRMC2 empty vector (control)	This study
walR1::p*GQP*	*walR*$_{\mathrm{T101M}}$ harbouring pRMC2 with *mupG-murQR* operon from *S. aureus* ATCC29213	This study
E. coli Stellar	Lab cloning *Escherichia coli* strain	Lab stock

[1] Corrigan, R. M. & Foster, T. J. An improved tetracycline-inducible expression vector for Staphylococcus aureus. *Plasmid* **61**, 126–129 (2009).

Supplemental Table 2 – Primers used in this study

Primer Name	Sequence	Purpose
p*GQP*-KpnI-F-Tag	TAAAATAAGCTTGATGGTACCAGGAGGATG**CATCACCATCACCATCAT**AGCAGCGG CATGATTAAACAAGGTTAC	Forward primer to amplify *mur* operon from *S. aureus* ATCC29123 with 6XHis tag
p*GQP* -EcoRI-R	TGTAAAACGACGGCCAGTGAATTCTTAATCACCCAACAAATCTGT	Reverse primer to amplify *mur* operon from *S. aureus* ATCC29123
pRMC2-F-Screening-MCS	ATCCCCTCGAGTTCATGAA	Forward primer for colony PCR-screening of insert
pRMC2-R-Screening-MCS	ATACTCATGTGCTGCAAGGC	Reverse primer for colony PCR-screening of insert

Supplemental Table 3) Single nucleotide polymorphisms identified in tunicamycin-resistant *S. aureus walR*1 mutants

Mutant	Nucleotide Change	Protein Change	Codon Position	Gene	Pan ID ¶	Function/Description
tun^{R1}	C → T	R → C	555	*walK*	SAUPAN000033000	Cell wall metabolism sensor histidine kinase
tun^{R2}	G → T	R → L	557	*walK*	SAUPAN000033000	Cell wall metabolism sensor histidine kinase
tun^{R3}	G → T	R → L	557	*walK*	SAUPAN000033000	Cell wall metabolism sensor histidine kinase
tun^{R4}	C → T	L → F	37	*walK*	SAUPAN000033000	Cell wall metabolism sensor histidine kinase
tun^{R5}	G → T	R → L	557	*walK*	SAUPAN000033000	Cell wall metabolism sensor histidine kinase
tun^{R6}	C → T	Truncation	160	*clpP*	SAUPAN002695000	ATP-dependent Clp protease proteolytic subunit

¶ Identification represented by AureoWiki PanGenome

Supplemental Table 4) Peptidoglycan fragments identified from *S. aureus* ATCC29213
The stem peptide consists of: L-Ala-D-Gln-L-Lys-D-Ala-(D-Ala). The glycine bridge is the number of glycines that form the interpeptide bridge of each fragment. Modifications observed were O-acetylation of N-acetyl muramic acid.

Peak	$[M+H]^+$	Molecular Formula	Stem peptide	Glycine Bridge	Modifications
1	897.4423	$C_{36}H_{65}N_{8}O_{18}^{+}$	4		
2	968.4828	$C_{39}H_{70}N_{9}O_{19}^{+}$	5		
3	1182.5484	$C_{46}H_{80}N_{13}O_{23}^{+}$	4	5	
4	1239.5711	$C_{48}H_{83}N_{14}O_{24}^{+}$	4	6	
5	1082.5220	$C_{43}H_{76}N_{11}O_{21}^{+}$	5	2	
6	1253.5870	$C_{49}H_{85}N_{14}O_{24}^{+}$	5	5	
7	1096.5368	$C_{44}H_{78}N_{11}O_{21}^{+}$	5	1 + 1xAla	
8	1295.6007	$C_{51}H_{87}N_{14}O_{25}^{+}$	5	5	+ O-acetyl
9	2189.0354	$C_{87}H_{150}N_{23}O_{42}^{+}$	5 + 4	6	
10	2417.1187	$C_{95}H_{162}N_{27}O_{46}^{+}$	5 + 4	10	

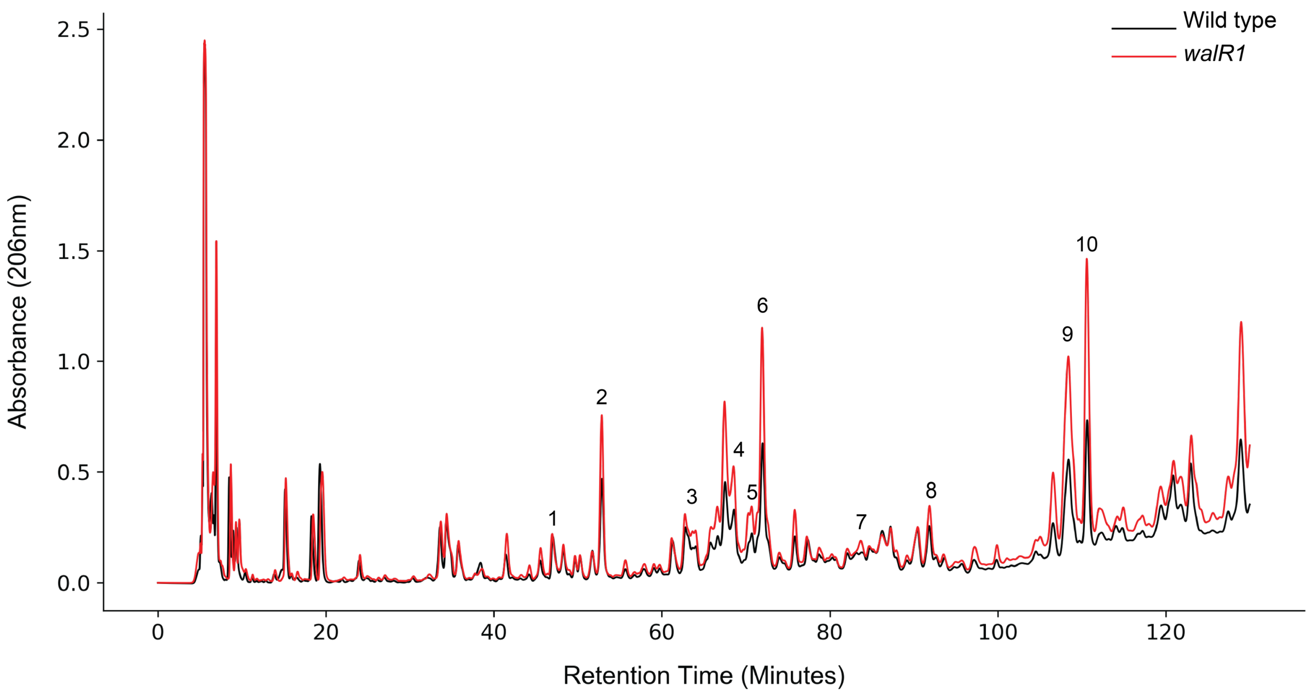

Supplemental Figure 1) Peptidoglycan composition is the same in *walR1*
Peptidoglycan fragments from wild type and *walR1* were purified and separated by high-performance liquid chromatography and fragments were detected by an absorbance of 206nm. Annotated peaks were identified according to Supplemental Table 4.
